# Supplementary material for: Role and mechanism of NCAPD3 in promoting malignant behaviors in gastric cancer
Source: Front Pharmacol. 2024 Apr 22;15:1341039. doi: 10.3389/fphar.2024.1341039 (PMC11070777; doi:10.3389/fphar.2024.1341039)
Supplement: Supplementary file 11 [file DataSheet2.ZIP › GSEA/immunologic/my_analysis.Gsea.1599462003817/GSE43863_NAIVE_VS_MEMORY_TFH_CD4_TCELL_D150_LCMV_UP.html]

Details for gene set GSE43863\_NAIVE\_VS\_MEMORY\_TFH\_CD4\_TCELL\_D150\_LCMV\_UP[GSEA]

|  || Dataset | filtered\_dataset.sample\_info.cls#WT\_versus\_NCAPD3\_MUT |
| Phenotype | sample\_info.cls#WT\_versus\_NCAPD3\_MUT |
| Upregulated in class | NCAPD3\_MUT |
| GeneSet | GSE43863\_NAIVE\_VS\_MEMORY\_TFH\_CD4\_TCELL\_D150\_LCMV\_UP |
| Enrichment Score (ES) | -0.57993084 |
| Normalized Enrichment Score (NES) | -2.5265455 |
| Nominal p-value | 0.0 |
| FDR q-value | 0.0137389805 |
| FWER p-Value | 0.09 |
Table: GSEA Results Summary

  

Fig 1: Enrichment plot: GSE43863\_NAIVE\_VS\_MEMORY\_TFH\_CD4\_TCELL\_D150\_LCMV\_UP      
 Profile of the Running ES Score & Positions of GeneSet Members on the Rank Ordered List

  

| SYMBOL | TITLE | RANK IN GENE LIST | RANK METRIC SCORE | RUNNING ES | CORE ENRICHMENT || 1 | 7763 | ZFAND5 | 26 | 1.006 | 0.0613 | No |
| 2 | 339559 | ZFP69 | 188 | 0.686 | 0.0004 | No |
| 3 | 901 | CCNG2 | 998 | -0.405 | -0.5477 | Yes |
| 4 | 55589 | BMP2K | 1017 | -0.417 | -0.5275 | Yes |
| 5 | 54676 | GTPBP2 | 1050 | -0.447 | -0.5149 | Yes |
| 6 | 54629 | FAM63B | 1110 | -0.483 | -0.5188 | Yes |
| 7 | 665 | BNIP3L | 1185 | -0.556 | -0.5277 | Yes |
| 8 | 153222 | CREBRF | 1187 | -0.559 | -0.4839 | Yes |
| 9 | 26999 | CYFIP2 | 1234 | -0.611 | -0.4684 | Yes |
| 10 | 7057 | THBS1 | 1242 | -0.620 | -0.4241 | Yes |
| 11 | 114907 | FBXO32 | 1248 | -0.625 | -0.3780 | Yes |
| 12 | 9262 | STK17B | 1268 | -0.659 | -0.3392 | Yes |
| 13 | 80853 | KDM7A | 1311 | -0.714 | -0.3126 | Yes |
| 14 | 2650 | GCNT1 | 1342 | -0.781 | -0.2720 | Yes |
| 15 | 54463 | FAM134B | 1356 | -0.815 | -0.2165 | Yes |
| 16 | 255394 | TCP11L2 | 1408 | -1.538 | -0.1308 | Yes |
| 17 | 760 | CA2 | 1410 | -1.654 | -0.0000 | Yes |
Table: GSEA details [plain text format]

  

Fig 2: GSE43863\_NAIVE\_VS\_MEMORY\_TFH\_CD4\_TCELL\_D150\_LCMV\_UP      
 Blue-Pink O' Gram in the Space of the Analyzed GeneSet

  

Fig 3: GSE43863\_NAIVE\_VS\_MEMORY\_TFH\_CD4\_TCELL\_D150\_LCMV\_UP: Random ES distribution      
 Gene set null distribution of ES for **GSE43863\_NAIVE\_VS\_MEMORY\_TFH\_CD4\_TCELL\_D150\_LCMV\_UP**
